# Investigation of Yellow Fever Virus at the Human–Animal Interface after a Zika Virus Outbreak in Midwest Brazil

**DOI:** 10.3390/microorganisms12030594

**Published:** 2024-03-16

**Authors:** Lis Alvarez Baranowski, Helver Gonçalves Dias, Débora Familiar-Macedo, Gilberto Sabino-Santos, Heitor Miraglia Herrera, Renata Dezengrini Slhessarenko, Flávia Barreto dos Santos, Alex Pauvolid-Corrêa

**Affiliations:** 1 Laboratório das Interações Vírus-Hospedeiros, Instituto Oswaldo Cruz, Fiocruz, Rio de Janeiro 21040-900, RJ, Brazil; lis.baranowski56@gmail.com (L.A.B.); helvergd@gmail.com (H.G.D.); deborafamiliar@gmail.com (D.F.-M.); 2 Department of Microbiology and Immunology, Tulane University School of Medicine, New Orleans, LO 70112, USA; gsabino@tulane.edu; 3 Centro de Pesquisa em Virologia, Faculdade de Medicina de Ribeirão Preto, Universidade de São Paulo, Ribeirão Preto 14049-900, SP, Brazil; 4 Laboratório de Biologia Parasitária, Programa de Pós-Graduação em Ciências Ambientais e Sustentabilidade Agropecuária, Universidade Católica Dom Bosco (UCDB), Campo Grande 79117-010, MS, Brazil; herrera@ucdb.br; 5 Laboratório de Virologia, Faculdade de Medicina, Universidade Federal de Mato Grosso (UFMT), Cuiabá 78060-900, MT, Brazil; renatadezengrini@yahoo.com.br; 6 Laboratório de Virologia Veterinária de Viçosa, Departamento de Veterinária, Universidade Federal de Viçosa (UFV), Viçosa 36570-900, MG, Brazil

**Keywords:** non-human primates, PRNT, state of Mato Grosso, state of Mato Grosso do Sul

## Abstract

Following the first report of zika virus in March 2015, Brazil experienced its largest sylvatic yellow fever outbreak between 2016 and 2019. This study aimed to investigate the circulation of yellow fever virus (YFV) in non-human primates (NHPs) and mosquitoes collected in urban parks and other metropolitan areas of midwest Brazil between 2017 and 2018. Whole blood samples from 80 NHPs, including 48 black-tailed marmosets (*Mico melanurus*) and 2332 mosquitoes from six different genera, were collected in the states of Mato Grosso (MT) and Mato Grosso do Sul (MS) and then tested for YFV by RT-qPCR. Additionally, 23 plasma samples of NHPs were tested for neutralizing antibodies for YFV by a plaque reduction neutralization test (PRNT). No YFV RNA or neutralizing antibodies for YFV were detected in NHPs and mosquitoes from MT and MS. The continuous monitoring of YFV circulation in different species of NHPs and vectors in urban areas is instrumental to quickly assess potentially unknown maintenance cycles of yellow fever at the human–animal interface in Brazil.

## 1. Introduction

The *Orthoflavivirus flavi* or yellow fever virus (YFV) is classified in the *Flaviviridae* family and *Orthoflavivirus* genus, and it was first isolated from the blood of a Ghanaian patient in 1927. Arbovirus transmission, such as YFV, primarily occurs through the blood meal of hematophagous arthropods in viremic hosts, followed by a second blood meal in a susceptible host [1,2].

In Brazil, the main vectors in YFV transmission cycles are *Haemagogus janthinomys* and *Hg. leucocelaenus* mosquitoes, and the main amplifying hosts are some species of non-human primates (NHPs), including howler monkeys (*Allouatta* spp.) [3]. Other species of mosquitoes, such as *Aedes albopictus*, *Ae. aegypti*, *Ae. scapularis*, *Ae. taeniorhynchus*, *Ae. serratus*, and *Psorophora ferox*, have also been found to be naturally infected by YFV in Brazil [2,4]. The urban cycle of YFV transmission involving humans as amplifying hosts and *Ae. aegypti* as vectors has not been reported in Brazil since the 1940s [2]. Given the ecological plasticity of *Ae. albopictus* in urban, rural, and forest environments in tropical countries, this species is believed to be able to act as an eventual bridge vector between sylvatic and urban environments. Experimental studies have shown that not only current populations of *Ae. albopictus* but also *Ae. aegypti* from different areas in Brazil present the capacity to transmit YFV, which raises concern about the potential reemergence of the urban cycle of yellow fever in the country [5].

Some species of NHPs are part of the sylvatic transmission cycles of YFV, acting as temporary amplifying hosts. In Brazil, NHP species of the genera *Alouatta*, *Callithrix*, *Aotus*, *Sapajus*, and *Atelles* are believed to be the most important groups [6,7,8]. The disease burden in NHPs varies among species; however, overall, the hemorrhagic disease is characterized by a sudden onset and rapid course, with viremia starting from the third day of infection [9,10]. The main signs of yellow fever in NHPs include fever, apathy, dehydration, vomiting, and liver and kidney failure. Neurological disorders are also seen in some NHPs during yellow fever epizootics [9].

Some species of neotropical NHPs are highly susceptible to YFV. In Brazil, epizootics of hemorrhagic fever in NHPs are normally seen in prior human cases. Because of that, surveillance of NHP epizootics is key to the early detection of YFV circulation to trigger the response of public health agencies. The most reported affected species of NHPs during epizootics of YFV in Brazil are howler monkeys (*Alouatta* spp.) [10]. However, whether they are the main amplifying hosts or whether there are other species that silently play a role in YFV amplification remains obscure. Even less information is available regarding what species of NHPs and vectors are involved in YFV transmission at the human–animal interface levels in the country.

Therefore, assessing the level of YFV exposure in different species of NHP and mosquito vectors is also of great relevance, especially in urban and peri-urban environments. The identification of mosquito and NHP species involved in sylvatic yellow fever in these areas in Brazil is crucial to guide vector control and assess the urbanization risk of yellow fever in the Americas. Here, we aimed to investigate potentially unreported sylvatic cycles of yellow fever at the human–animal interface, evaluating the exposure to YFV of NHPs and mosquitoes collected mainly in urban parks in MT and MS between 2017 and 2018.

## 2. Materials and Methods

As part of a larger study, the ethical aspects of the collections have been previously addressed [11]. Briefly, collections were approved by the Animal Ethics Committee from the Federal University of Mato Grosso (UFMT-23108.169037-06/23/2016-24) and Dom Bosco Catholic University (UCDB-001-03/23/2017; 005-04/24/2017) and authorized by Brazilian environment state agencies (SEMA 201624/2017 and IMASUL 61405959/2016) and by the Instituto Chico Mendes of Biodiversity Conservation (ICMBio) (MS-57450-1, 56912-1; MT-19838-6, 54728-1).

**Figure 1:**
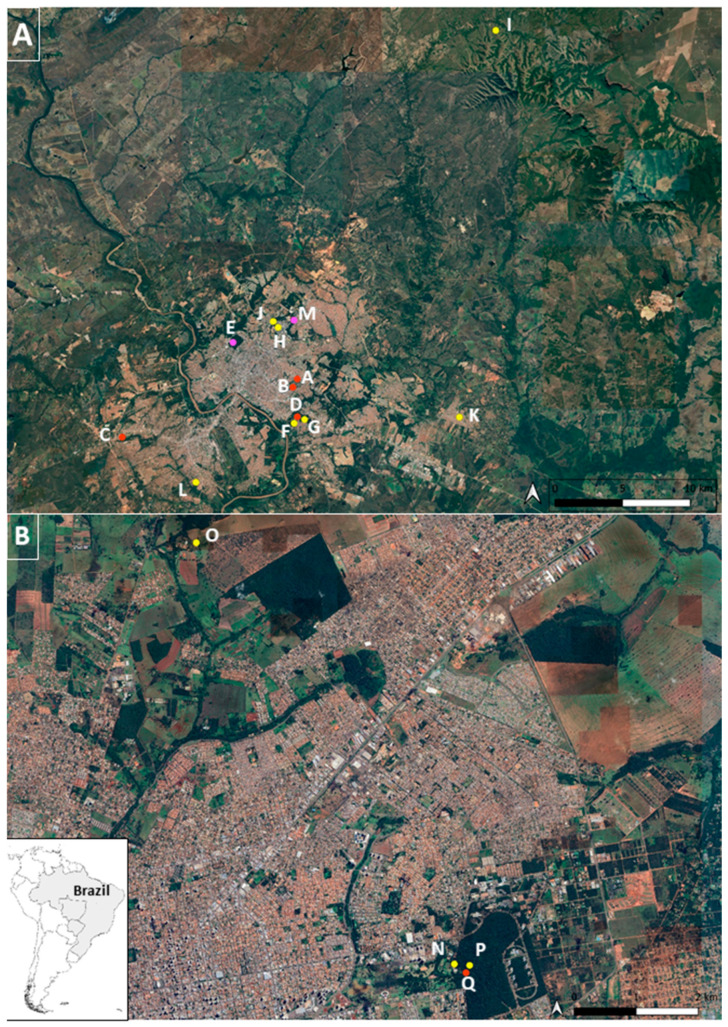
Sampling sites within the cities of Cuiabá, MT (picture (**A**)) and Campo Grande, MS (picture (**B**)); A–Q sampling subsites. Red dotes mark subsites where non-human primates were sampled; yellow dotes mark subsites where mosquitoes were collected; pink dotes mark subsites where both mosquitoes and non-human primates were sampled.

NHP and vectors were collected in 17 sampling areas, with 13 in the metropolitan area of MT (A-M) and 4 in the metropolitan area of MS (N-Q). Sampling sites included local neighborhoods, riverside communities, hotels, urban parks, zoos, veterinary hospitals, animal breeders associations, water parks, environmental police battalions, and a wildlife rehabilitation center. Most sampling sites were located within the city of Cuiabá in MT and within the city of Campo Grande in MS. Blood samples of NHPs were collected from free-ranging individuals captured in urban parks of MT and also from captive individuals from both states (Figure 1).

**Figure 2:**
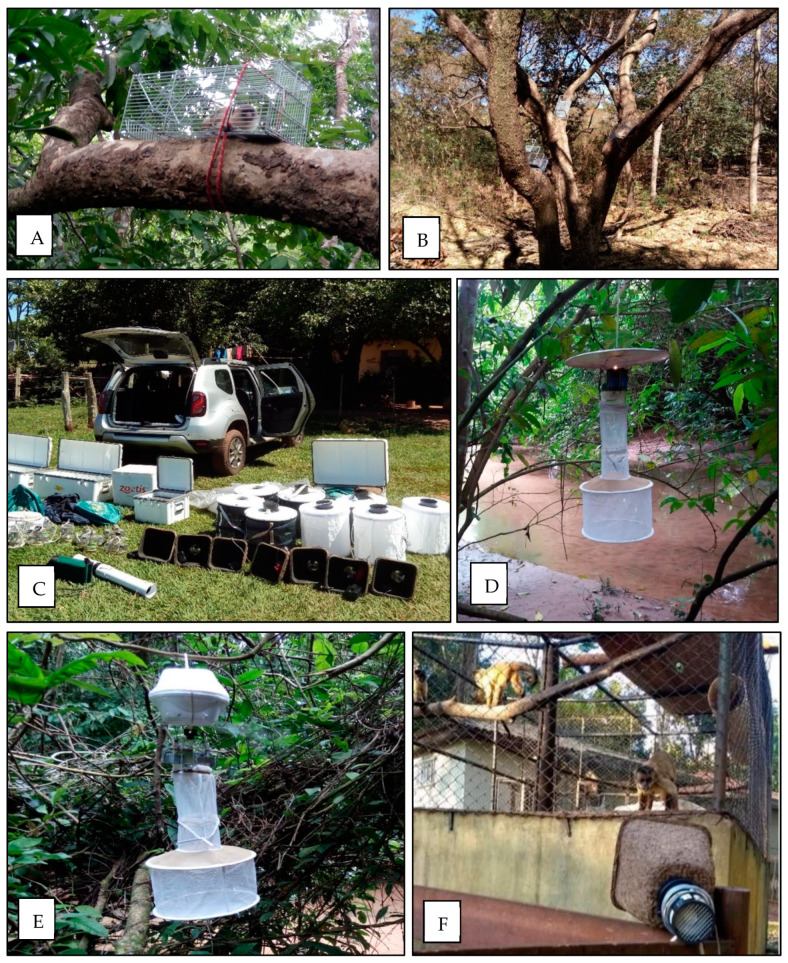
Traps used for collection of non-human primates and mosquitoes in MS and MT during 2017–2018. (**A**,**B**) Tomahawk traps distributed in trees during non-human prmates collection in MT. (**C**–**F**) BG-Sentinel Traps, CDC light, CO_2_ CDC, and resting traps, respectively (credit: Alex Pauvolid-Corrêa and Helver Dias).

Collections were performed during four campaigns of approximately 2 to 3 weeks each, including samplings during the dry season (April to May and July to August 2017) and the rainy season (October to November 2017 and February to March 2018) and were carried out simultaneously by teams of professionals, who took turns. At each sampling site, animals were captured for approximately 4 consecutive days. Initially, 4 to 6 transects were opened at each site, with the placement of 1 trap every 20 m. The traps used were of the Tomahawk type (45 × 17.5 × 15 cm) (Figure 2), with attractive baits made from bananas, vanilla essence, cereals, and macerated sardines. Briefly, venous blood samples were collected by puncture of the femoral vein in the plexus arteriovenous of the inguinal region from NHPs (*n* = 80) were collected in tubes containing sodium citrate anticoagulant and included specimens from *Sapajus cay* (*n* = 13), *Callithrix jacchus* (*n* = 4), *Callithrix penicillata* (*n* = 1), *Mico melanurus* (*n* = 48), *Aotus lemurinus* (*n* = 2), *Alouatta caraya* (*n* = 5), *Sapajus apella* (*n* = 5), and *Ateles marginatus* (*n* = 2). Detailed information on the sample collections has been published elsewhere [11].

It is important to highlight the conservation status of some of the NHP specimens captured for this investigation. The species *Alouatta caraya*, *Ateles marginatus*, and *Mico melanurus* are considered by the Red List of Threatened Species as threatened, while the species *Sapajus cay* and *Aotus lemurinus* are classified as vulnerable, and the specimens *Callithrix jacchus*, *Callithrix penicillata*, and *Sapajus apella* as of least concern (Table 1).

In the field, mosquitoes were collected using backpack aspirators or handheld Insectazooka or Prokopak aspirators, CDC light traps, sometimes baited with CO_2_, BG-Sentinel Traps^TM^ baited with human lure, octanol, or CO_2_, and resting traps aiming to capture engorged mosquitoes (Figure 2). All collected arthropods were morphologically screened, and all mosquitoes (Culicidae) were placed in cryovials with up to 25 specimens. Cryovials were then taken to the laboratory and kept in −80C freezers. In the laboratory, the sampled mosquitoes were separated into two groups, one including non-engorged female mosquitoes (*n* = 2258) and another one including engorged female mosquitoes (*n* = 74). All mosquito specimens were then morphologically identified to species over a chill plate by entomologists. Genera found among non-engorged mosquitoes included *Aedes* (*n* = 138), *Haemagogus* (*n* = 87), *Sabethes* (*n* = 16), *Psorophora* (*n* = 211), *Culex* (*n* = 1805), and *Mansonia* (*n* = 1). The engorged mosquitoes included *Aedes* (*n* = 65), *Haemagogus* (*n* = 8), and *Sabethes* (*n* = 1).

Identified mosquitoes were separated by pools of up to 25 individuals according to species, gender, type of trap in which they were caught, and the day and site of collection. Engorged mosquitoes were also captured, their individual information was recorded, and they were kept with one specimen per cryovial. The identification of the mosquito specimens collected was carried out by the Hematozoan Transmitting Mosquito Laboratory (IOC/FIOCRUZ), using the dichotomous keys described by Forattini and collaborators [12,13].

The non-engorged mosquitoes, grouped in pools of 25 specimens, were placed in 2 mL screw-cap tubes containing 5 mm glass grinding beads (Sigma-Aldrich, Saint Louis, MO, USA) and 550 μL of 199 media with antibiotics. Screw-cap tubes were then submitted to agitation at 25 Hertz for 4 min (TissueLyser, Qiagen, Hilden, Germany). Engorged mosquitoes had their abdomens separated and homogenized as described for the non-engorged specimens. The remaining carcasses of the non-engorged mosquitoes were kept in a separate tube for further analysis.

Biosafety levels 2 and 3 laboratories were used for RNA extraction. Commercial ZR-96 Viral RNA kit (Zymo Research, Irvine, CA, USA) was used for NHP and non-engorged mosquito samples. Engorged mosquitoes had RNA extracted using the commercial Invitrogen™ PureLink™ Viral RNA/DNA kit protocol (Invitrogen, Waltham, MA, USA).

Active YFV infection was investigated in plasma samples of NHP and mosquitoes by RT-qPCR, as described by Domingo et al. [14], which uses the Taqman System designed for the amplification of a fragment of 82 base pairs.

Additionally, the presence of neutralizing antibodies specific for YFV in NHP plasma samples was also investigated by PRNT in VERO CCL-81 cells. A wild-type YFV strain (strain BR4408/08) isolated from an *Allouata caraya* captured in 2008 in Brazil was provided by the Laboratory of Hematozoan Transmitters (LATHEMA, Fiocruz). Due to plasma volume limitation, 23 out of the 80 NHPs, including *Sapajus cay* (*n* = 12), *Alouatta caraya* (*n* = 5), *Callithrix jacchus* (*n* = 3), *Callithrix penicillata* (*n* = 1), *Mico melanurus* (*n* = 1), and *Ateles marginatus* (*n* = 1) were tested. All NHP plasma was heat-inactivated (56C for 30 min) and then tested for its ability to neutralize YFV plaque formation.

Briefly, inactivated plasma samples diluted 1:5 were incubated for one hour at 37 °C with 5% CO_2_ atmosphere with an equal volume of viral suspension containing 30–100 plaque forming units (PFU), resulting in a final dilution of 1:10. At the end of incubation, 100 µL of the mixture was inoculated into VERO cell monolayers with 750,000 cells per well (1 mL) in 6-well plates. A second incubation for the same period and conditions was performed for viral adsorption. At the end of the second incubation, 3 mL of a 0.5% agarose solution and cell maintenance medium was applied before the third and final incubation, this time for 4 days, under the same temperature and CO_2_ conditions. After that, 3 mL of an agarose solution with neutral red dye was added and incubated for another 24 h for the final reading. The neutralizing antibody titer was determined as the inverse of the serum dilution at which a 90% or greater reduction in the number of PFU in the viral suspension control used in the assay was observed.

## 3. Results

NHP individuals were captured in different locations. In MS, all 22 individuals were sampled in an animal rehabilitation center, including *Sapajus cay* (*n* = 13), *Alouatta caraya* (*n* = 4), *Callithrix jacchus* (*n* = 4), and *Callithrix penicillata* (*n* = 1). These animals were brought from different parts of the state and kept in captivity for rehabilitation. Some were found injured or sick, on highways, or recovered from animal trafficking.

**Figure 3:**
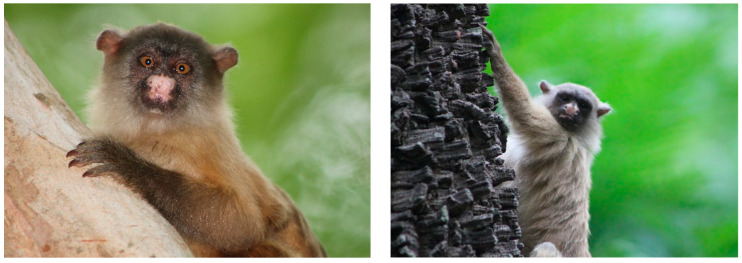
Specimens of black-tailed marmosets (*Mico melanurus*) captured in Cuiabá, 2017–2018 (credit: Alex Pauvolid-Corrêa).

In MT, a total of 58 NHPs, including captive and free-ranging individuals, were sampled. Ten individuals were captive including *Sapajus apella* (*n* = 5), *Alouatta caraya* (*n* = 1), *Ateles marginatus* (*n* = 2), and *Aotus lemurinus* (*n* = 2). These animals were sampled in zoos, veterinary hospitals, and environmental police battalions and some of them were kept in captivity for rehabilitation. Forty-eight free-ranging black-tailed marmosets (*Mico melanurus*), the most common species, were captured in urban parks (Figure 3). 

All non-engorged mosquitoes were collected using four trap types. The handheld aspirator collected 1018 individuals (45.2%) classified to five genera, whereas the BG collected 445 (19.7%) individuals classified to four genera. The use of the CDC light trap resulted in the collection of 107 (4.7%) specimens classified to four genera. On the other hand, the use of the CDC light with the addition of CO_2_, as attractive bait, resulted in the collection of 473 (20.9%) individuals classified to four genera. The CDC resting trap collected a total of 215 (9.5%) mosquitoes classified to only two genera (Table 2).

Engorged mosquitoes (*n* = 74) were also collected, and the majority of them were from the genus *Aedes* (*n* = 65), followed by *Haemagogus* (*n* = 8), with only one individual from the genus *Sabethes*. From those, 22 specimens (29.7%) did not have information on the trap used and location. By using the handheld aspirator, a total of 37 (50.0%) engorged mosquitoes were collected, while using the BG and CDC with CO_2_, 6 (8.1%) specimens were collected, respectively. The CDC light trap collected two (2.8%) individuals and the resting trap only one individual (Table 3).

In this study, not all mosquitoes collected were identified to the species level. Some damaged individuals could only be identified at the genus level. Among *Aedes*, *Ae. aegypti* (51.7%), and *Ae. albopictus* (27.6%) were the most frequent species identified. Among *Haemagogus*, *Hg. janthinomys* (61.0%) and *Hg. leucocelaenus* (25.3%) were the only two species identified. Regarding *Sabethes*, three specimens of *Sa. glaucodaemon* and 12 specimens of *Sa. purpureus* were found. Twelve individuals were identified to *Sabethes* spp. All individuals identified to *Culex* and the only specimen identified to *Mansonia* were not classified at the species level as well. Among *Psorophora*, *Ps. dimidiata* was the most common species (Table 4).

All NHPs (*n* = 80), engorged mosquitoes (*n* = 74), and non-engorged mosquitoes (*n* = 2258) tested by RT-qPCR were negative for YFV RNA (Ct > 38).

To investigate the previous exposure of NHPs to YFV by determining specific neutralizing antibodies, plasma sample of 23 NHPs identified to six different species in MT (*n* = 20) and MS (*n* = 3) were tested (Table 5).

Since MT and MS have confirmed circulation of other flaviviruses, PRNT_90_ was conducted using a conservative criterion of seropositivity. To be considered seropositive, neutralization of at least 90% of the average of PFU observed in the viral control was used. All NHP plasma samples tested presented PRNT_90_ titers <10 and were therefore considered seronegative for YFV.

## 4. Discussion

Yellow fever is of great importance in Brazil, not only for its medical relevance but also for its potential impact on wildlife conservation. It has been historically considered endemic mainly in the Amazon region. However, the number of human cases and epizootics in NHPs has increased in several other regions of the country [15].

In our investigation, eight species of NHP were investigated. These species present different geographic distributions, behavioral aspects, sizes, and for most of these, the relevance to the YFV sylvatic cycle in Brazil remains to be fully determined. All 80 NHPs were sampled through active surveillance. Free-ranging individuals were presumably healthy and presented normal behavior. This active surveillance aimed to gather information not only about the health of the NHPs but also their exposure to YFV locally. The negative results obtained here corroborate the absence of epizootics among the sampled individuals. Interestingly, an investigation for zika virus in wildlife conducted in the same region, period and using the same methods found no evidence of zika virus infection in the NHPs. These findings indicate that, despite the circulation in human population of both orthoflaviviruses in MT and MS, the NHPs tested at the interface human-animal were not exposed [11]. 

The genera *Allouata*, *Callithrix*, *Sapajus*, and *Atelles* are considered the most important amplifying hosts of YFV [15,16]. However, the majority of individuals tested in the present study were *Mico melanurus* (*n* = 48), found not only in MT and MS but also in the states of Amazonas and Rondônia [17,18]. In the present investigation, black-tailed marmosets were only found and captured in urban parks in the city of Cuiabá.

According to Camera and Canale, *Mico melanurus* has an arboreal activity similar to that of the YFV-transmitting mosquitoes (*Haemagogus* spp. and *Sabethes* spp.), with an acrodendrophilic habitat [17,18]. However, unlike the genera *Atelles* and *Allouata*, which are present in high canopies and rarely come down to the ground, the black-tailed marmoset tends to remain in the lower forest strata, typically below 10 m [17,18]. The absence of exposure evidence in *Mico melanurus* may indicate that this species is not a preferred blood feeding source for vectors.

The PRNT, considered the gold standard by the World Health Organization for analyzing the response to vaccination or natural infection by YFV, is a highly specific serological test that determines the presence of specific neutralizing antibodies [19]. A main advantage of the assay is the ability to differentiate infection among different orthoflaviviruses, which can be of particular interest in serosurveys in Brazil, where dozens of orthoflaviviruses circulate [20].

One limitation of the present study is that, from 80 NHPs captured, only 23 were submitted to PRNT, due to the small plasma volume collected from some individuals. Serological tests are particularly informative, as the presence of antibodies indicate previous exposure. Here, all the NHPs tested were seronegative, indicating those animals were likely not exposed to YFV.

The largest YFV epidemic/epizootic, that started in Brazil in 2014–2016 and reached the southeast region, the most populated region in the country, raised several hypotheses about its path. One possibility would be the transmission of YFV from northern to southeast Brazil facilitated by environmental disturbance, through vector mosquitoes and NHPs, perhaps involving NHP species not commonly involved in YFV cycles of transmission [21]. Despite increasing environmental degradation, some ecological corridors present in the Brazilian Atlantic Forest have expanded, promoting a recovery of fauna and flora, contributing to the creation of a more conducive environment for the arrival and maintenance of YFV in other areas than the northern region [4]. The midwest region of Brazil, where NHPs and vectors were sampled and tested, is located between these two regions.

A total of 2258 non-engorged and 74 engorged mosquitoes of the genera *Aedes*, *Haemagogus*, *Sabethes*, *Psorophora*, *Culex*, and *Mansonia* were collected in MT and MS and tested for YFV RNA. Although all mosquitoes were negative for YFV infection, the collection of these specimens provided valuable information on the ecology and dispersion of different species in the region. Specimens of *Ae. albopictus* and *Ae. aegypti*, considered a YFV bridge vector and an urban cycle vector, respectively, were collected in our investigation. *Ae. albopictus* is an opportunistic species, being present in peri-urban and wild areas, with wide feeding habits and found close to the ground [22]. Although *Ae. albopictus* does not share the same environment as acrodendrophilic NHP, it is common for YFV-infected and sick NHPs to descend to the ground, thus becoming a possible blood feeding source [5]. Unlike *Ae. albopictus*, *Ae. aegypti* has endophilic and anthropophilic habits, also being considered an important species with high potential for YFV transmission [23]. In our investigation, 203 specimens of the genus *Aedes* were collected, including 105 (51.7%) *Ae. aegypti* and 56 (27.6%) *Ae. albopictus.* Among the other species found was *Aedes serratus*, a species already found naturally infected by YFV in Brazil. Although *Psorophora ferox*, also found infected by YFV elsewhere, was not captured in this investigation, five other species of the genus *Psorophora* were reported.

The capture of *Hg. janthinomys* and *Hg. leucocelaenus* specimens in urban parks commonly frequented by visitors in midwest Brazil can be of particular concern. The activity of the two main YFV vector species [24] associated with the reduction in yellow fever vaccination coverage highlights the continuous risk of yellow fever outbreaks and potential re-urbanization in Brazil.

Among the limitations of the study is the unequal distribution of the number of individuals by genera of non-engorged and engorged mosquitoes. While the non-engorged mosquitoes were all females, most of the specimens (79.9%) were represented only by the genus *Culex*, while only one individual of the genus *Mansonia* was collected. The total number of engorged mosquitoes was much smaller compared to the non-engorged, and the sample also had only one representative of the genus *Sabethes*. It was also not possible to identify all mosquitoes collected at the species level.

Despite the negative results of the molecular analyses conducted with all mosquitoes, the collection of specimens provided rich information on the activity and dispersion of different species in the region. The continuous surveillance of YFV circulation in different species of NHP and vectors is an important tool to quickly assess the potential maintenance cycles of YFV at the human–animal interface in Brazil. Moreover, further research is needed to elucidate the intricacies of YFV circulation and potential amplifying hosts, considering the interplay between vertebrates and mosquitoes in the ecological context.

## Figures and Tables

**Table 1:** Non-human primate species sampled and tested for YFV in Midwest Brazil.

Non-Human Primate Species	N	Common Name	Conservation Status
*Sapajus cay*	13	Azara’s Capuchin	Vulnerable
*Alouatta caraya*	5	Howler Monkey	Threatened
*Aotus lemurinus*	2	Colombian Night Monkey	Vulnerable
*Ateles marginatus*	2	White-cheeked Spider Monkey	Threatened
*Callithrix jacchus*	4	Common Marmoset	Least Concern
*Callithrix penicillata*	1	Black-penciled Marmoset	Least Concern
*Mico melanurus*	48	Black-tailed Marmoset	Threatened
*Sapajus apella*	5	Black-capped Capuchin	Least Concern
TOTAL	80		

**Table 2:** Distribution of non-engorged mosquitoes (*n* = 2258) collected according to the trap and collection site.

Trap Type	City	*Aedes* (*n*)	*Culex* (*n*)	*Haemagogus* (*n*)	*Sabethes* (*n*)	*Mansonia* (*n*)	*Psorophora* (*n*)	Total (*n*)
Handheld aspirator	Cuiabá	103	821	33	1	0	5	
	Campo Grande	0	0	48	7	0	0	
	Total	103	821	81	8	0	5	1018
BG	Cuiabá	0	433	0	0	0	0	
	Campo Grande	1	4	0	6	1	0	
	Total	1	437	0	6	1	0	445
CDC light	Cuiabá	10	65	4	0	0	1	
	Campo Grande	10	4	0	0	0	13	
	Total	20	69	4	0	0	14	107
CDC with CO_2_	Cuiabá	6	224	2	0	0	0	
	Campo Grande	7	40	0	2	0	193	
	Total	13	264	2	2	0	193	473
Resting	Cuiabá	0	213	0	0	0	0	
	Campo Grande	1	1	0	0	0	0	
	Total	1	214	0	0	0	0	215
Total		138	1805	87	16	1	212	2258

**Table 3:** Distribution of engorged mosquitoes (*n* = 74) collected according to the trap and collection site.

Trap Type	City, State	*Aedes*	*Haemagogus*	*Sabethes*	Total
Handheld aspirator	Cuiabá, MT	27	4	0	
	Campo Grande, MS	2	4	0	
	Total	29	8	0	37
BG	Cuiabá, MT	4	0	0	
	Campo Grande, MS	2	0	0	
	Total	6	0	0	6
CDC light	Cuiabá, MT	1	0	0	
	Campo Grande, MS	1	0	0	
	Total	2	0	0	2
CDC with CO_2_	Cuiabá, MT	0	0	0	
	Campo Grande, MS	5	0	1	
	Total	5	0	1	6
Resting	Cuiabá, MT	1	0	0	
	Campo Grande, MS	0	0	0	
	Total	1	0	0	1
No location	Total	22	0	0	22
Total		65	8	1	74

**Table 4:** Distribution of engorged and non-engorged mosquitoes by species identification.

Culicidae Species	N	%
*Aedes aegypti*	105	51.7
*Aedes albopictus*	56	27.6
*Aedes augustivittatus*	2	1.0
*Aedes fulvithorax*	4	2.0
*Aedes* spp.	36	17.7
Total	203	100
*Culex* spp.	1805	100
Total	1805	100
*Haemagogus janthinomys*	58	61.0
*Haemagogus leucocelaenus*	24	25.3
*Haemagogus* spp.	13	13.7
Total	95	100
*Psorophora albigenu*	1	0.5
*Psorophora cilipes*	37	17.5
*Psorophora cingulata*	31	14.7
*Psorophora dimidiata*	108	51.2
*Psorophora lanei*	22	10.4
*Psorophora* spp.	12	5.7
Total	211	100
*Mansonia* sp.	1	100
Total	1	100
*Sabethes glaucodaemon*	3	17.6
*Sabethes purpureus*	2	11.8
*Sabethes* spp.	12	70.6
Total	17	100
Total	2332	100

**Table 5:** Distribution of NHP species from MT and MS submitted to PRNT_90_ for YFV.

		Campo Grande, MS		Cuiabá, MT			
NHP	Popular Name	N	%	N	%	PRNT_90_ Titer	Total
*Alouatta caraya*	Black-and-Gold Howler Monkey	4	20	1	33.3	<10	5
*Ateles marginatus*	White-whiskered Spider Monkey	1	5	0	0	<10	1
*Callithrix jacchus*	Common Marmoset	3	15	0	0	<10	3
*Callithrix penicillata*	Black-tufted-ear Marmoset	0	0	1	33.3	<10	1
*Mico melanurus*	Black-tailed Marmoset	1	5	0	0	<10	1
*Sapajus cay*	Hooded Capuchin	11	55	1	33.3	<10	12
Total		20	100%	3	100%		23

## Data Availability

All the data available are included in the manuscript.
